# The Outcome and Impact of Academic Cancer Clinical Trials with Participation from Canadian Sites (2015–2024)

**DOI:** 10.3390/cancers17244009

**Published:** 2025-12-16

**Authors:** Rebecca Y. Xu, Diana Kato, Victoria Percival, James Schoales, Stephen Sundquist, Raisa Chowdhury, Gregory R. Pond, Janet E. Dancey

**Affiliations:** 1 Canadian Cancer Clinical Trials Network, Toronto, ON M5G 0A3, Canada; dkato@oicr.on.ca (D.K.); jschoales@oicr.on.ca (J.S.); ssundquist@oicr.on.ca (S.S.); rchowdhury@oicr.on.ca (R.C.); 2 Faculty of Law, University of Toronto, Toronto, ON M5S 1A1, Canada; victoria.percival@mail.utoronto.ca; 3 Department of Oncology, McMaster University, Hamilton, ON L8S 4L8, Canada; gpond@mcmaster.ca; 4 Department of Oncology, Canadian Cancer Trials Group, Queen’s University, Kingston, ON K7L 3N6, Canada

**Keywords:** academic cancer clinical trials, Canadian Cancer Clinical Trials Network (3CTN), phase III trials, rare cancers, trial outcomes, trial reporting, cancer research impact, evidence-based oncology, clinical practice guidelines, cancer guidelines

## Abstract

Background/Objectives: Academically sponsored cancer clinical trials (ACCTs) are essential for advancing patient-centered care, particularly in areas underserved by commercial research. The Canadian Cancer Clinical Trials Network (3CTN) was established to support high-quality multi-center ACCTs through coordinated infrastructure and funding. Over ten years, funders provided an average of CAD 4.3 million annually (~CAD 0.11 per capita), primarily from federal and provincial sources. This study evaluates the outcomes and impact of trials supported by 3CTN between 2015 and 2024. Methods: We conducted a descriptive analysis of 350 ACCTs that stopped recruiting and had primary completion dates within the study period. Trial characteristics, results, publication rates, and incorporation into clinical guidelines were assessed using registry data, peer-reviewed publications, and structured searches of oncology guidelines. Results: Among these 350 closed trials, 116 were Phase III studies. Of these, 36% were incorporated into clinical practice guidelines, and 7% were likely to be incorporated. Overall, 81% of trials were published in journals, and 45% posted results in public registries. Trials addressed diverse cancer types, with notable contributions in rare cancers and vulnerable populations. Conclusions: 3CTN-supported ACCTs had high completion and reporting rates, with substantial influence on clinical practice. These findings highlight how sustained infrastructure and modest public investment can deliver meaningful improvements in cancer care and inform evidence-based policy.

## Simple Summary

Academically sponsored cancer clinical trials are important for improving care, especially for patients with rare cancers or unique needs. These trials often explore questions that matter most to patients and clinicians. In Canada, the Canadian Cancer Clinical Trial Network (3CTN) supported hundreds of such trials over the past decade through a coordinated national infrastructure and public funding. Funders provided about CAD 4.3 million annually (~CAD 0.11 per capita), mainly from public sources. This study assessed outputs and impact by examining publication of results and incorporation into practice guidelines. Completion and publication rates were high, and 36% of Phase III closed trials led to changes in clinical practice, showing how sustained investment in academic-sponsored trials and trial infrastructure can drive meaningful improvements in cancer outcomes for patients and clinicians while informing policy and funding decisions.

## 1. Introduction

Clinical trials are essential for advancing scientific knowledge, improving cancer care, and providing patients with access to innovative therapies [1]. Among these, cancer clinical trials led by academic investigators and cooperative clinical trial groups might be distinct in their purpose from industry-sponsored trials, which are often driven by product development pipelines. Although the objectives may sometimes overlap, academic-led trials often focus on underexplored clinical questions, rare cancers, real-world effectiveness of interventions, unmet clinical needs, or patient-centered outcomes such as quality of life assessments [2,3].

The influence of academic cancer clinical trials (ACCTs) on clinical practice has been significant, driving advances in screening, prevention, and treatment approaches, including combination chemotherapy regimens, multimodality therapies, and treatments of rare cancers. In the United States, 45.1% of National Cancer Institute Clinical Trials Network (NCTN)-supported trials contributed to clinical guidelines or drug approvals [4]. In Canada, 62.8% of Phase III trials sponsored by the Canadian Cancer Trials Group (CCTG) were cited in clinical guidelines [5]. Despite their clinical and scientific value, ACCTs can face added resource constraints due to funding limitations and lower budgets, as well as accrual challenges [6,7,8]. A lack of core funding to support clinical trial unit operations and increased expectations for institutional cost recovery have tended to favour participation in industry-sponsored trials [9].

In response to observed year-over-year declines in the number of new ACCTs, site participation, and accruals to open trials in Canada [2], the Canadian Cancer Clinical Trials Network (3CTN, Network) was established in 2014 through public funding. Currently, there are 38 adult and 13 pediatric member cancer centres and hospitals located in eight Canadian provinces. Member centres, organized into local-regional nodes overseen by the larger tertiary cancer centres, commit to participating in a portfolio of ACCTs (the 3CTN ‘Portfolio’). All trials are multicenter, have undergone peer review, and have some funding to support trial activities. Participating members report their Portfolio ACCT performance on a quarterly basis; members also participate in collaborative initiatives to improve the quality and efficient performance of trials opened locally. A central Coordinating Centre serves as the administrative and communications hub, providing systems support, oversight of data reporting processes, project coordination, and a comprehensive, accessible library of data informatics, best practice tools, and resources.

To help ensure a robust Canadian ACCT ecosystem, portfolio trial candidates are required to be peer-reviewed, academic-sponsored, interventional, and multicentered. The 3CTN governance and operating frameworks enable the creation of relevant strategic priorities. Provincial core funding support for member centre trial units in each region creates capacity to achieve aligned objectives, while incentive funding from the national funder can be earned by centres for surpassing performance targets aligned with the network’s overall strategic goals for accrual and rapid trial activation. Over the course of ten years, funders provided an average of approximately CAD 4.3 million annually (~CAD 0.11 per capita; Canada ~40 M population), primarily from federal and provincial public sources. For context, the UK’s National Cancer Research Network in 2004 invested approximately GBP 20 million annually [2] (~CAD 46 M at historical rates; ~CAD 0.77 per capita; UK ~59.8 M population), underscoring that Canada’s investment is modest compared with international benchmarks despite being the largest and most sustained investment in cancer clinical trials conducted in Canada.

Since its inception, there have been over 850 trials included in the 3CTN Portfolio. With member trial performance data, 3CTN holds one of the most comprehensive ACCT datasets in Canada. While Canada contributes strongly to global oncology research, there has not been a systematic assessment of ACCT outcomes and impact at the national level. This study addresses that gap.

The goals of this study were to assess the outcomes and impact of ACCTs supported by 3CTN between 2015 and 2024. Specifically, we

- Identified trends and gaps in completed trials, including patterns in evidence dissemination and translation into clinical practice.
- Assessed the contributions of 3CTN-supported trials to peer-reviewed publications and clinical treatment guidelines.

## 2. Materials and Methods

### 2.1. Data Sources and Inclusion Criteria

**Figure 1:**
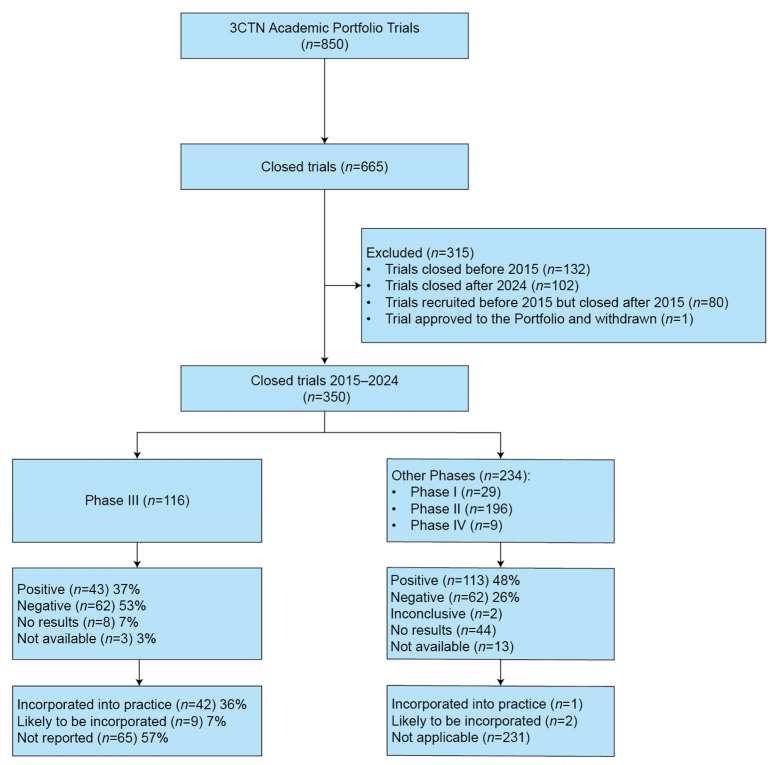
Data selection process for included trials (2015–2024). Data lock: 9 October 2025.

**Figure A1:**
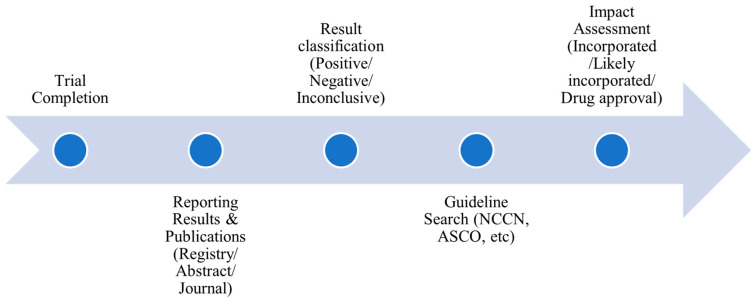
Flow diagram of impact tracking process for practice change assessment.

This exploratory descriptive analysis used data from the 3CTN ACCT Portfolio. Eligible trials were academic-sponsored, interventional, multicenter, peer-reviewed, and involved Canadian sites. Closed trials included those active in Canada that stopped recruitment with primary completion dates between 2015 and 2024. Trials withdrawn prior to recruitment or terminated after activation but before planned completion were classified as prematurely completed to capture challenges in trial conduct. This timeframe aligns with the period following the establishment of the 3CTN Portfolio and allows time for trial completion and publication. Data was extracted from the Portfolio database, clincialtrials.gov, and PubMed. The data selection process is illustrated in Figure 1. Phase III trials are presented separately as the primary source for practice-changing evidence; Phases I, II, and IV are combined to provide context. The workflow used to assess practice-changing impact is detailed in Appendix A Figure A1.

### 2.2. Operational Definitions and Classification

To ensure consistency and reproducibility, an impact framework was applied with standard definitions available on the 3CTN website [10]; see Appendix A Table A1 for relevant definition details. The following key operational definitions were applied for trial status and results:

- Closed: Trials that stopped recruiting, including those with statuses of closed to recruitment, terminated, or completed.
- Closed to Recruitment: Trials that stopped enrolling participants but may still be in follow-up
- Completed: Trials that reached planned accrual and primary endpoint analysis.
- Prematurely Completed: Trials withdrawn before recruitment or terminated before planned accrual (e.g., due to poor accrual or toxicity).

Trial results were classified based on primary outcome reporting:

- Positive: Results are reported in the literature and demonstrate a positive primary outcome.
- Negative: Results are reported in the literature and demonstrate a negative primary outcome.
- Inconclusive: Direction or significance of the primary outcome could not be determined from available data, including trials terminated before reaching target accrual.

When no results were reported, we used the following to monitor them:

- Not available: No peer-reviewed results were found, and the primary study completion date is <24 months ago.
- No results: No peer-reviewed results were found, and the primary study completion date is ≥24 months ago.
- Pending final publication: No final, peer-reviewed results for the entire study population, but interim or subgroup results are available, or a peer-reviewed source explicitly states that results are pending or expected.

### 2.3. Assessment of Study Results and Practice Impact

Closed trials were reviewed at least annually for registry updates and publications, with additional reviews triggered by new relevant publications to classify the trial results as positive, negative, or inconclusive.

Practice impact was assessed primarily for Phase III trials through structured annual searches of major North American and European oncology guidelines, supplemented by ad hoc reviews when new publications emerged (Section Oncology Guidelines Reviewed for Practice Change Assessment for guideline list). Clinical impact was categorized as follows:

- Incorporated into Practice Guidelines: results cited in the evidence that informed a guideline recommendation (not just cited as background evidence).
- Likely to be Incorporated into Practice Guidelines: Peer-reviewed sources explicitly indicated that trial results are currently influencing clinical decisions or expected to appear in future guideline updates.

### 2.4. Data Quality Control

Manual searches were supplemented by a machine learning tool called “Trials to Publications”, developed by University of Illinois College of Medicine, which was used intermittently to validate linkages between trials and resulting publications [11]. Although not central to the analysis, these tools supported quality control and may offer future opportunities for automation.

## 3. Results

### 3.1. Trial Characteristics

Between 2015 and 2024, 350 ACCTs closed (41% of the 3CTN Portfolio), including 116 Phase III trials. Of the total, 31 (9%) closed prematurely before planned completion, with the most often cited reason being poor accrual (Appendix A Table A2). Most trials were randomized, evaluated drug therapies, and involved diverse cancer types and settings. Trials evaluating precision medicine strategies were common (68%), while 33% addressed rare cancers and 22% involved vulnerable populations (see Appendix A Table A3 for the Characteristics of the 3CTN Portfolio Closed Trials 2015–2024).

### 3.2. Trial Outcomes

**Figure 2:**
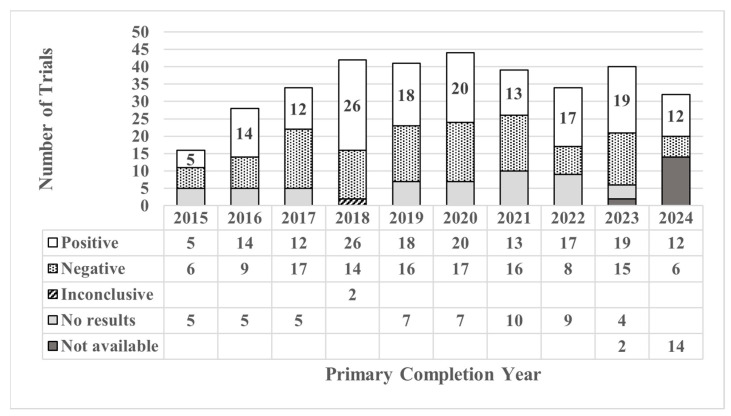
Annual distribution of study results for closed trials (*N* = 350).

Of the 350 closed trials that were reviewed for results compared with primary outcomes, 156 (44%) were classified as positive, 124 (35%) were negative, and a small portion were inconclusive (1%) or had no results reported (20%). Of the 68 trials with no results reported, 39 (57%) were closed between 2021 and 2024. Figure 2 highlights a peak in trial completions in 2020, followed by a decline in subsequent years, which may reflect broader trends such as the impact of the COVID-19 pandemic on trial performance, capacity, or changes in research priorities among some sponsors and site institutions.

#### 3.2.1. Reporting and Publication Rates

Rates for posting of portfolio trial results in registries, as well as publication in scientific journals, are summarized in Table 1. Among all closed trials, 45% (159/350) had reported results posted in a public registry, primarily on ClinicalTrials.gov. 81% (282/350) published their findings as abstracts or full articles, with 117 trials being published in high-impact journals, defined as those ranked within the top 10% based on 2022 CiteScores. The top three journals were the Journal of Clinical Oncology, JAMA Oncology, and the New England Journal of Medicine.

#### 3.2.2. Practice Guideline Incorporation

**Figure 3:**
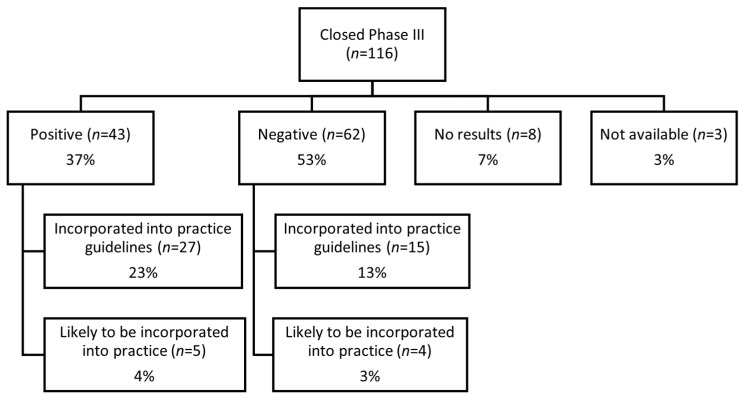
Phase III trial result breakdown with distribution of practice-changing trials incorporated into guidelines.

Of the 116 closed Phase III ACCTs, 36% (42/116) were incorporated into practice guidelines, and 7% (9/116) were marked as “likely to be incorporated into practice”, as they were cited in practice guidelines for future consideration or contributed to new drug approvals. Among 43 positive Phase III clinical trials, 32 (74.4%) were either already or likely to be incorporated into practice. Both positive and negative results contributed to evidence-based care (see Figure 3 for the distribution of the practice-changing trials). Although Phase III trials were the primary focus of the review, a small portion of Phase II and Phase IV trials were also found to have led to practice changes (see Figure 1).

#### 3.2.3. Sponsorship and Practice-Changing Impact

Among the 116 closed Phase III trials in the 3CTN Portfolio, the two largest academic sponsors were the U.S. National Cancer Institute (NCI) and the Canadian Cancer Trials Group (CCTG). NCI sponsored 67 adult and pediatric trials (58%), while CCTG sponsored 39 trials (33%), reflecting the important role of cooperative groups in supporting academic oncology research.

Importantly, 42 trials (36%) led to changes in clinical practice through incorporation into guidelines or contribution to drug approval decisions. Of these, 31 trials (74%) were NCI-funded, highlighting a strong association between NCI sponsorship and practice-changing outcomes.

### 3.3. Recruitment Contributions from Network Sites

Based on the reported accrual from the 3CTN database, 3CTN member sites contributed 17% of the global recruitment to closed trials. Contributions grew over time, climbing to 33% in 2024, highlighting the Network’s role in the accrual success of ACCTs (Table 2).

For Phase III trials specifically, 3CTN sites contributed 9% to the overall global recruitment (Table 3) and 6.6% of recruitment to practice-changing trials (Table 4).

## 4. Discussion

Our findings demonstrate that ACCTs supported by 3CTN were associated with high completion and reporting rates, as well as contributed substantially to evidence-based cancer care. Portfolio trials reflect a broad spectrum of high-quality research, with a notable emphasis on rare cancers, supportive care, and vulnerable populations (i.e., pediatric or elderly patients), areas often underserved by industry-sponsored trials [12]. The incorporation of 36% of Phase III trials into clinical guidelines or contribution to drug approvals compares favorably with existing literature. For example, Elimova et al. reported that 20% of positive Phase III trials published in high-impact journals between 1990 and 2010 were incorporated into practice guidelines [13]. While not directly comparable due to differences in inclusion criteria and timeframe, our analysis, which encompasses both positive and negative trials, shows that academic trials supported by 3CTN have had a substantial and diverse impact on clinical practice. Publication bias remains a concern; although negative trials were often published, trials with positive results are more likely to be published more quickly and reported more completely than trials with negative or no results [14,15,16]. Historically, negative trials have faced publication delays, taking a median of 6.5 years versus 4.3 years for positive trials [17]. Delayed and incomplete reporting underscores the need for continued efforts to ensure accurate and timely reporting and mechanisms such as funder mandates and institutional incentives to enhance compliance. Practice-changing contributions were often seen through repurposing, optimization of treatment regimens, or patient management strategies and were most prevalent in studies involving hematologic, breast, genitourinary, and gynecologic cancers. A selection of notable practice-changing trials is listed in Appendix A Table A4.

To make these impacts visible and accessible to stakeholders, 3CTN developed an interactive outcome and publication search board for all portfolio trials closed to recruitment. Built using Microsoft Power BI, this publicly available tool enhances transparency and enables users to explore trial-related publications, posted results, and impacts on clinical guidelines. It serves as a centralized resource for Network stakeholders. The full dataset and dashboard are available on the 3CTN website [18].

Canadian participation in NCI-sponsored trials has remained consistently high over the past decade, reflecting strong international collaboration and the strategic alignment between Canadian trial units and U.S. cooperative groups. This underscores the pivotal role of NCI sponsorship in generating practice-changing evidence and highlights Canada’s meaningful contribution to these global efforts.

Despite persistent challenges and limitations affecting our national trial system [9,19], the number of Canadian-led trials and recruitment to NCI-funded studies have increased. At the same time, Canadian-led trials have demonstrated leadership in areas often underserved by industry-sponsored research, including rare cancers, supportive care, systemic therapies, and radiotherapy. These contributions reflect the strength of Canada’s academic trial ecosystem and the value of sustained support for investigator-led research.

A total of 282 (81%) closed trials had results reported at the time of review, including trials with inconclusive findings. Of the 350 trials, 145 (41%) closed between 2021 and 2024, and 39 (11%) had no results or results not yet available (<24 months post-closure), suggesting that reporting delays may be due to ongoing analysis or publication processes, including editorial decisions. Overall, improvements in recruitment and outcomes reporting rates for closed trials occurred during the 3CTN period, suggesting a contributing role for the Network. 3CTN’s supportive infrastructure and initiatives designed to overcome barriers to ACCT conduct in Canada were aimed at increasing the participation of clinicians and patients and enabling the completion of trials and subsequent publication. The substantial impact achieved with a relatively modest annual budget demonstrates the value of a coordinated national program to support academic cancer clinical trials.

Journal publication rates may improve through enhancing recruitment, timeliness of reporting, and adoption of innovative trial methods. Over time, the use of adaptive and master protocols for rare cancer histology or molecularly defined subgroups increased, as did decentralized trial conduct and digital tools for data collection. Trials testing precision medicine strategies also became more common. In addition, incentivizing researchers and journals to publish all trials, including those with negative outcomes. Future opportunities to improve trial accrual and completion include the applied use of artificial intelligence to support the translation of patient information materials into multiple languages to promote equitable access and improve the screening of medical records against complex inclusion criteria.

There are several limitations to this study. Our analysis depended on trial performance data reported by 3CTN sites and available registry data, which varied in completeness. Data was reviewed and validated to the extent possible. Publication and guideline searches, while extensive and labor-intensive, may have missed citations due to inconsistent and/or delayed referencing in guidelines. Use of natural language processing (NLP) and machine learning tools was incorporated for supplemental data validation activities and illustrated the potential for automating and enhancing these tasks. However, further validation will be required before routine use. In addition, recently closed trials may not yet have been included in practice guidelines, underestimating impact. Our use of publications and citations to assess impact, while important, does not fully capture broader policy or patient-level outcomes. Despite recent efforts to promote publication of negative results [20,21,22], further improvements are needed to ensure that all trial outcomes contribute to scientific knowledge and resource optimization [23]. While improvements in high completion and reporting rates occurred during the 3CTN period, the descriptive design of this study does not provide insights into causal relationships or predictive factors. Broader global trends in trial transparency and evolving regulatory requirements are among other factors that may have influenced observed outcomes.

## 5. Conclusions

3CTN-supported ACCTs have high completion and reporting rates, with substantial influence on practice guidelines and patient care. A focus on vulnerable populations, supportive care, and identifying better treatments for patients with rare cancers highlights the value of ACCTs. 3CTN and member cancer center trial units require long-term infrastructure support to sustain and expand upon performance improvements and beneficial impacts realized to date. Recognizing the essential role of the Network in supporting these achievements is critical. Coupled with sustained investments in academic cancer trials, system innovation, performance improvement, and strategic priorities for equitable trial access, Canada can leverage its strengths and position itself as a global leader in advancing patient-centered oncology research. Doing so is not only a scientific imperative but also a strategic opportunity to shape the future of cancer care for all Canadians.

## Figures and Tables

**Table 1:** Closed trial results reported by phase.

Phase	All Trials	Reported in Registry	Journal Publication
Phase I	29	2 (7%)	21 (72%)
Phase II	196	79 (40%)	148 (76%)
Phase III	116	76 (66%)	105 (91%)
Phase IV	9	2 (22%)	8 (89%)
Total	350	159 (45%)	282 (81%)

**Table 2:** Annual patient recruitment to closed portfolio trials (2015–2024).

Year Trial Closed	Number of Trials	Sample Size	Global Recruitment *	3CTN Sites Recruitment	3CTN Sites Contribution (%)
2015	16	2930	2158	181	8%
2016	28	4406	2894	836	29%
2017	34	8861	7596	859	11%
2018	42	14,873	14,231	3134	22%
2019	41	14,536	16,554	2595	16%
2020	44	20,217	20,458	2962	14%
2021	39	11,886	11,221	2312	21%
2022	34	25,381	18,903	2894	15%
2023	40	18,777	17,821	2521	14%
2024	32	11,553	8223	2681	33%
Total	350	133,420	120,059	20,975	17%
Median	36.5	13,211	12,726	2558	15%
IQR	8.25	8267	9751.5	1618.5	7%

* Global recruitment taken from clinicaltrials.gov record. Missing global recruitment data for eight trials closed (one in 2015, one in 2020, two in 2021, one in 2023, and three in 2024). IQR = interquartile range. 3CTN site contribution = 3CTN site recruitment/global recruitment.

**Table 3:** 3CTN member site contribution to Phase III ACCTs closed (2015–2024).

Study Results	Number of Trials	Global Recruitment	3CTN Sites Recruitment
Negative	62 (53%)	52,999	3763 (7%)
No results	8 (7%)	1500	355 (24%)
Not available	3 (3%)	385	139 (36%)
Positive	43 (37%)	37,679	3771 (10%)
Total	116	92,563	8028 (9%)

**Table 4:** Canadian ACCTs in guidelines and 3CTN global recruitment contribution.

Study Results	Number of Trials *	Global Recruitment	3CTN Sites Recruitment
Negative	15	211,518	958 (4.5%)
Positive	28	32,826	2593 (7.1%)
Total	43	54,007	3551 (6.6%)

* Including phase III and IV practice changing trials of this period.

## Data Availability

The data presented in this study are contained within this article and also in the 3CTN Portfolio Outcomes and Publications Search Board: https://3ctn.ca/outcomes-and-publication-search/.

## Abbreviations

The following abbreviations are used in this manuscript:

3CTN	Canadian Cancer Clinical Trials Network
ACCT	Academic Cancer Clinical Trials
ALL	Acute Lymphoblastic Leukemia
ASCO	American Society of Clinical Oncology
ASTRO	American Society for Radiation Oncology
CCO	Cancer Care Ontario
CCTG	Canadian Cancer Trials Group
CNS	Central Nervous System
COG	Children’s Oncology Group
COVID-19	Coronavirus Disease 2019
CMA	Canadian Medical Association
DSMB	Data Safety Monitoring Board
EANM	European Association of Nuclear Medicine
ESMO	European Society for Medical Oncology
GU	Genitourinary
ML-DS	Myeloid Leukemia of Down Syndrome
NCCN	National Comprehensive Cancer Network
OICR	Ontario Institute for Cancer Research
SIOPE	European Society for Paediatric Oncology
SNMMI	Society of Nuclear Medicine and Molecular Imaging

## Appendix A

**Table A1:** Key terminology and definitions used to assess impact and practice-changing trials.

Term	Definition
**Study Results**	
Positive	Results are reported in the literature and demonstrate a positive primary outcome:
	For phase III trials, a positive primary outcome was defined as a statistically significant (*p* < 0.05, or per the study’s prespecified threshold) and favorable effect for the experimental treatment compared with a placebo or active comparator.
	For earlier phase trials, a positive primary outcome was defined as sufficient efficacy/safety evidence to justify progression to a later phase trial, as stated by the authors.
Negative	Results are reported in the literature and demonstrate a negative primary outcome, meaning
	For phase III trials, a negative primary outcome was defined as an effect that is not statistically significant or is statistically significant but favors the control arm. For earlier phase trials, a negative primary outcome was defined as insufficient evidence to justify progression to a later phase trial.
Inconclusive	Results were classified as inconclusive when the direction or significance of the primary outcome could not be determined from the available data, including cases where the study was prematurely terminated prior to reaching target accrual.
No Results Reported	**Not available**: No peer-reviewed results were found, and the primary study completion date is <24 months ago.
	**No results**: No peer-reviewed results were found, and the primary study completion date is ≥24 months ago
	**Pending final publication:** No final, peer-reviewed results for the entire study population, but interim or subgroup results are available, or a peer-reviewed source explicitly states that results are pending or expected.
**Practice Changing**	
Incorporated into Practice	Trials were considered incorporated if their results were cited as evidence supporting a North American or European guideline recommendation, regardless of whether the finding was positive or negative. If a trial was cited only to highlight its contribution to an unresolved clinical question or to support evidence from other studies, without informing a specific recommendation, it was not considered incorporated.
Likely to be Incorporated into Practice	The trial’s findings were not yet included in guideline updates or revisions, but a peer-reviewed source explicitly states that the results are informing clinical decisions, currently influencing practice, or are likely to be incorporated into clinical guidelines in the future. FDA or Health Canada approval often precedes or triggers guideline updates, so regulatory approval was considered a strong indicator of likely incorporation.
**Other Key Terms**	
Rare Cancers	Refers to adult cancer that occurs in <6 out of 100,000 people each year using the European standard; pediatric cancers occur in <2 out of 1,000,000 children each year.
Vulnerable Populations	Pediatric (<18), AYA (15–39), Elderly (>70)
Precision Medicine	Focuses on matching cancer treatments to the specific genetic and molecular features of a patient’s tumor, often using advanced testing to identify mutations or biomarkers that can be targeted by specific therapies.

### Oncology Guidelines Reviewed for Practice Change Assessment

Guideline inclusion was assessed by reviewing recommendations from the following major oncology, general medical, and specialty-specific international bodies:

- National Comprehensive Cancer Network (NCCN)
  ○ via both the JNCCN journal (https://jnccn.org).
  ○ and the official NCCN guideline PDFs (https://www.nccn.org/professionals/physician_gls).
- American Society of Clinical Oncology (ASCO)
  ○ via https://asco.org/practice-patients/guidelines.
  ○ and ASCO Publications (https://ascopubs.org) for joint or collaborative guidelines updates.
- American Society for Radiation Oncology (ASTRO) Guidelines
- European Society for Paediatric Oncology (SIOP Europe or SIOPE)
- International Childhood Liver Tumors Strategy Group—for pediatric liver cancer
- Cancer Care Ontario (CCO)
- American Urological Association Guidelines
- European Association of Urology—for prostate and bladder cancer.
- European Society for Radiotherapy and Oncology
- European Society for Medical Oncology (ESMO)—for European oncology practice
- European Association of Neuro-Oncology—for neuro-oncology
- Canadian Medical Association (CMA)
- Trip Database-https://www.tripdatabase.com—aggregates global guidelines.
- EANM/SNMMI Joint Guidelines for trials involving diagnostic imaging. These guidelines are published by the European Association of Nuclear Medicine and the Society of Nuclear Medicine and Molecular Imaging.
- The American Society for Transplantation and Cellular Therapy Practice Guidelines
- Society of American Gastrointestinal and Endoscopic Surgeons—GI Surgery for all GI Surgeons

Note: Searches will include both full guideline documents and peer-reviewed guideline updates to ensure that trials cited in collaborative or journal-based recommendations (e.g., ASCO–CCO updates, JNCCN articles) are captured. In the case of multiple guidelines being mentioned, we will cite the primary North American guidelines, such as NCCN, ASCO, and ASTRO.

**Table A2:** Terminated and withdrawn trials by phase (2015–2024).

Reasons Cited for Termination	Phase I	Phase II	Phase III	Phase IV	Grand Total
Drug company decision		2	1		3
DSMB review			1		1
Lack of funding		1			1
Negative study		2			2
Poor accrual	3	14	1	1	19
Staffing issues				1	1
Unacceptable Toxicity			1		1
Unknown		2			2
COVID-19 pandemic		1			1
Total	3	22	4	2	31

**Table A3:** Characteristics of closed 3CTN portfolio trials 2015–2024.

Trial Characteristics	Analysis	Phase III	Overall
Number of trials	N	116	350
Study Phase	I	-	29 (8%)
	II	-	196 (56%)
	III	116	116 (33%)
	IV	-	9 (3%)
Disease Site	Bone	1 (1%)	5 (1%)
	Brain/CNS	8 (7%)	19 (5%)
	Breast	16 (14%)	49 (14%)
	Gastrointestinal	11 (9%)	32 (9%)
	Genito-Urinary	17 (15%)	56 (16%)
	Gynecological	12 (10%)	25 (7%)
	Head and neck	3 (3%)	11 (3%)
	Hematology	23 (20%)	55 (16%)
	Lung	10 (9%)	32 (9%)
	Neuroblastoma	1 (1%)	7 (2%)
	Other	9 (8%)	43 (12%)
	Sarcoma	4 (3%)	9 (3%)
	Skin/Melanoma	1 (1%)	7 (2%)
Country of Sponsor	Canada	33 (28%)	192 (55%)
	United States	71 (61%)	142 (41%)
	Other	13 (11%)	16 (5%)
Sponsor	NCI (USA)	67 (58%)	123 (35%)
	CCTG	39 (33%)	87 (25%)
	COG	21 (6%)	41 (12%)
Special Interest	Lifestyle interventions	4 (3%)	10 (3%)
	Novel therapy	9 (8%)	69 (20%)
	Rare cancer setting	39 (33%)	109 (31%)
	Vulnerable populations	26 (22%)	71 (20%)
	Precision medicine	80 (68%)	80 (23%)
Interventions	Behavioral	3 (3%)	13 (4%)
	Drug	75 (64%)	232 (66%)
	Device	3 (3%)	8 (2%)
	Radiation	39 (33%)	82 (23%)
	Procedure	0 (0%)	58 (17%)
	Biological	24 (21%)	51 (15%)
Type of Design	Basket Trial	0	2
	Platform Trial	1	5
	Umbrella Trial	0	1
	Low-complexity method	1	5
	Multiple steps	13	22
Completion status	Closed to recruitment	60 (52%)	129 (37%)
	Completed	52 (45%)	190 (54%)
	Prematurely completed (terminated or withdrawn)	4 (3%)	31 (9%)

**Table A4:** Notable Phase III Trials in the 3CTN Portfolio. A full list of trials that have publications and outcomes can be found on the 3CTN website: https://3ctn.ca/outcomes-and-publication-search/). The following is only a selection of those that were open and closed between 2015 and 2024.

Trial	Practice-Defining Trial Outcome	Disease Site	Active Recruitment	NCT Number	Recruitment Contribution (%)	Publication	Practice Guidelines Changed
NRG-CC001	Recommended HA-WBRT plus memantine to reduce neurocognitive decline in patients with brain metastases.	Brain Metastases	2016–2018	NCT02360215	8.9% (46/518)	[24]	NCCN [25]
(CCTG) MA.36/Olympia	Adjuvant Olaparib is recommended for HER2-negative, BRCA-mutated early breast cancer with residual disease after neoadjuvant chemotherapy, based on Olympia trial results. FDA approves Olaparib for adjuvant treatment of high-risk early breast cancer.	Breast	2015–2019	NCT02032823	1.9% (35/1837)	[26]	NCCN [27]
OCOG-2016-PETABC	The PETABC trial supported the guideline recommendation for using 18F-FDG PET/CT in staging stage IIB–III breast cancer, showing improved detection of stage IV disease and influencing treatment decisions.	Breast	2016–2022	NCT02751710	100% (369/369)	[28]	EJNMMI [29]
(CCTG) MA.37/PALLAS	The PALLAS trial showed no benefit of adjuvant Palbociclib, leading to guideline recommendations against its use in early breast cancer.	Breast	2017–2025	NCT02513394	2.6% (152/5796)	[30]	NCCN [31]
(EORTC) 1333-GUCG/PEACE III	Combining radium-223 with Enzalutamide for mCRPC showed improved progression-free survival and potential overall survival benefit.	GU/Prostate	2018–2023	NCT02194842	4.5% (20/446)	[32]	NCCN [33]
GOG–0275	For low-risk gestational trophoblastic neoplasia, supportive methotrexate and actinomycin-D are effective first-line single-agent therapies.	Gyne/Gestational Trophoblastic	2015–2017	NCT01535053	3.5% (2/57)	[34]	NCCN [35]
(CCTG) ENC.1/NRG-GY018 / MK-3475-868	Pembrolizumab plus chemotherapy as a new standard for advanced or recurrent endometrial cancer, regardless of mismatch repair status, led to FDA approval and guideline inclusion.	Gyne/Endometrial	2021–2022	NCT03914612	3.7% (30/813)	[36]	NCCN [37]
(CCTG) CLC.2/Alliance A041202	Ibrutinib was superior to BendamustineB–rituximab for older patients with untreated CLL, supporting guideline recommendations and FDA-approved frontline use.	Chronic Lymphocytic Leukemia (CLL)	2015–2016	NCT01886872	7.9% (43/547)	[38]	NCCN [39]
(COG) AHOD1331	Use of Brentuximab Vedotin with AVE-PC for high-risk pediatric Hodgkin lymphoma showed superior efficacy and reduced need for radiation.	Hodgkin lymphoma	2015–2019	NCT02166463	6.5% (39/600)	[40]	NCCN [41]
(COG) AALL1331	For relapsed pediatric B-ALL, Blinatumomab was a treatment option despite early trial termination and no significant difference in disease-free survival.	B-ALL	2015–2019	NCT02101853	7.9% (53/669)	[42]	NCCN [43]
(COG) AAML1531	For ML-DS, supporting risk-based treatment and use of HD-AraC to improve outcomes in standard-risk patients.	ML-DS	2016–2022	NCT02521493	5.4% (15/280)	[44]	SIOP Europe [45]
(CCTG) ALC.4 (ECOG E1910)	Adding Blinatumomab to consolidation chemotherapy for newly diagnosed B-lineage ALL, improving overall survival, and establishing a new standard for BCR::ABL1-negative patients.	ALL	2017–2019	NCT02003222	1.8% (9/488)	[46]	NCCN [47]
(CCTG) HDC.1/SWOG S1826	Nivolumab + AVD as first-line treatment for advanced-stage Hodgkin lymphoma, showing better progression-free survival than BV + AVD; now a Category 1 recommendation in NCCN guidelines.	Hodgkin Lymphoma	2021–2022	NCT03907488	1.8% (18/994)	[48]	NCCN [49]
(EORTC) STRASS	There is no overall benefit of preoperative radiotherapy for retroperitoneal sarcoma, but it is supported for selective use in Liposarcoma.	Sarcoma	2015–2017	NCT01344018	4.5% (12/266)	[50]	NCCN [51]
(CCTG) SRC.7/Alliance A091105	Sorafenib significantly improved progression-free survival in desmoid tumors and is now recommended in NCCN guidelines as a systemic therapy option.	Sarcoma	2015–2016	NCT02066181	5.7% (5/87)	[52]	NCCN [51]
(CCTG) SC.24	The SC.24 trial showed stereotactic body radiotherapy improved pain control over conventional radiotherapy for spinal metastases; it is cited in Ontario guidelines for spine SBRT planning and delivery.	Spinal Metastases	2015–2019	NCT02512965	76.4% (175/229)	[53]	CCO [54]
